# Inquiry of the Practice of Leadership in Chengguan: A Study Based on the Ethnographic Research of Z City

**DOI:** 10.3389/fpsyg.2022.857043

**Published:** 2022-06-21

**Authors:** Hua Li, Yifan Li

**Affiliations:** ^1^Department of Social and Ecological Civilization, Shandong Academy of Governance, Jinan, China; ^2^School of Management, Xi'an Jiaotong University, Xi'an, China

**Keywords:** leadership, organization, behavior, ethnographic, Chengguan

## Abstract

Chengguan, the Urban Administrative Law Enforcement Bureau, has been criticized for its dismal public image and poor job performance. Based on an ethnographic case study in Z City, we analyzed the type of leadership that results in passive work performance in Chengguan, and we examined whether any leadership style can be used to improve Chengguan's image and performance. We developed a new leadership ontology of CNP (Cognition–Normalization–Performance) on this foundation, and ethnographic research was conducted in three phases: leader's cognition, followers' normalization, and organization performance. Several implications were drawn. Leader selection should be cautious and can be improved by studying the leader's traits and behavior. This is done by investigating candidates' leadership career paths, trait characteristics, motive profiles, and other qualities. It is useful to change leaders by strengthening followers' unity and cohesion by setting up a labor union, youth federation, women's federation, and other groups. A leader should be selected among individuals who have completed leadership training as opposed to appointing one from outside the organization. On the one hand, the superior should help to improve the leadership environment (context), supervise problems in the organization's operation and performance, and track changes over time. On the other hand, the leader can also provide the followers with a flexible and adaptive place of work.

## Introduction

Members of China's Urban Administrative Law Enforcement Bureau, better known as the Chengguan, are tasked with supporting police in enforcing “non-criminal administrative regulations” such as traffic, environment, and sanitation rules, mainly regarding floating vendors and street merchants to maintain the appearance, environment, and sanitation of cities. Since its establishment in the late 1990's, the Chengguan has performed far below expectations, and its image has been tarnished in testimonies given by the media and the masses.

As a newly established government organization, Chengguan is a system that utilizes, transforms, and combines human, capital, material, ideational, and other resources into a specific, problem-solving whole that aims to satisfy particular human demands (e.g., maintain city order). The structure interacts with other systems of human activities and resources (e.g., police) in a given city. Similar to police departments, Chengguan is also a rule-bonded, frontline law enforcement organization whose task is fulfilled through its frontline officers' activities; it also belongs to a street-level bureau.

In general, work performance is a complex and multifaceted construct (O'Toole and Meier, 2015). As for Chengguan, the focus of their frontline exercises is task performance and conscientiousness (Hassan et al., 2019). Task performance refers to the execution of work duties (Williams and Anderson, 1991); for Chengguan, these tasks include management of floating vendors and street merchants, maintenance of city sanitation, and other responsibilities.

In addition, conscientiousness, as an organization-focused citizenship behavior, refers to officers' voluntary acceptance and adherence to Chengguan's policies, rules, regulations, and procedures, even when the officers are not closely supervised or monitored (Organ et al., 2006). Conscientiousness means that the officers realize that they represent and are part of the Chengguan organization. Conscientious officers are reliable and trustworthy, and they abide by the organization's regulations, rules, norms, and practices. In doing so, they avoid engaging in behaviors that may undermine the organization's interests and tarnish its reputation (Bateman and Organ, 1983). However, many people have reported being slapped, beaten, held on the ground, and even thrown onto the streets from vehicles by some Chengguan officers. Vendors have also spoken of having their possessions confiscated and being made to pay for the return of their belongings by members of the Chengguan bureau. These results have helped create a bad reputation for Chengguan in some cities. In view of this, in the context of Chengguan's law enforcement responsibilities, being conscientious is equal in importance to completion of specified work duties.

While the duties of the Chengguan force are completed by the frontline officers on the streets, the task performance and conscientiousness of Chengguan as a whole depend on its leaders. As everything flows from leadership, the success or failure of human endeavors largely depends on the kind of leadership that is present. Leadership is always one of the most relevant aspects in an organizational context.

The leaders of Chengguan are key to the organization. Chengguan's operation cannot be separated from the leader's decision-making and control. The leadership style, method, and behavior of Chengguan's management affect the psychology and behavior of the staff, thereby directly shaping the performance of the members' activities in an authoritarian context. Moreover, the staff of Chengguan can adjust their own thinking and behavior by experiencing the internalized culture and, to some extent, can shape their director's leadership.

Leadership, in general and by nature, is an influencing process (Stogdill, 1950; Katz and Kahn, 1978; Cohen, 1984; Hersey and Blanchard, 1988; Maxwell, 2011), as leadership occurs when two or more people are involved: a leader and one or more followers. Moreover, such people are trying to attain given objectives. The quality of a leader's performance is directly related to the management of their followers' performance.

These general principles apply to the topic of this research and bring us to our research questions: What is the logic of leadership in influencing an organization? What forms of leadership existed in Chengguan and how effective were they? What practical and scholarly insights can be obtained in terms of improving leadership effectiveness?

The logic is that establishing effective leadership helps to drive improvements in teamwork, quality, safety, transformation, and innovation in contemporary organizations (Greenfield, 2007). The existing experience management, scientific management, human-oriented management, values leadership, ethical leadership, and so on may be serious challenges for organizations, but transforming them can reap great benefits.

This ethnographic research, which explores seven leaders of the Z Chengguan bureau, analyzes factors that shape the leaders' cognition, such as their characteristics, behavior premises [McGregor (1960) Theory X or Theory Y], and leadership experience; the outcomes of the leaders' decisions regarding Chengguan employees' work conduct; and the effects of these factors on the organization's performance. Over the course of the study, this research developed a joint Chengguan Leadership ontology and analysis methodology, Cognition–Normalization–Performance (CNP), and some practical suggestions are offered based on the research findings.

The subsequent sections are as follows: the literature review examines the research on leadership theories, and the main task is to answer the first questions presented in this article; next, the methodologies are described; then, ethnographic case studies on the city are presented with reference to Fairholm (2010) leadership perspective model and Van's five-category leadership theory; the key findings and theoretical framework are presented next; a discussion of the results and their implications is presented in the last section.

## Literature Review

### The Leadership Concept and Its Function

Leadership is one of the most widely discussed and debated issues in both academia and industry (Avolio et al., 2003; Bennis, 2007), but the definition of leadership remains ambiguous. Since the 1950's, researchers have been providing dozens of definitions of leadership. A selection is introduced here. Stogdill (1950) proposed that leadership may be considered as the process (act) of influencing the activities of an organized group in its efforts toward goal setting and goal achievement. Katz and Kahn (1978) view leadership as “the influential increment over and above mechanical compliance with the routine directives of the organization.” Leadership is also defined as the process of influencing the activities of an individual or a group toward goal achievement in a given situation (Hersey and Blanchard, 1988). In fact, it seems that there are almost as many definitions of leadership as there are scholars who have attempted to define the concept (Bass, 1990). Bass (1990) holds the opinion that leadership is an interaction between two or more members of a group. Leadership occurs when one group member modifies the motivation or competencies of others in the group. Any member of the group can exhibit some amount of leadership. As to the ontology of leadership, Pardey (2007) states that cognitive skills continue to have strong associations with various aspects of leadership and its effectiveness. Leadership involves lifting of a person's vision to higher sights, raising of a person's performance to a higher standard, and building of a person's personality beyond its normal limitations. While the definition varies among researchers, there are some commonalities. For example, Mackenzie and Barnes (2008) found some underlying consensus in major leadership approaches and a total of eight consensus items, which range from “leadership is a good thing and more of it is better” to “leadership is a type of holonomic process.”

The core and focus for leadership are that it is the process of exerting an influence with which a person enlists the aid and support of others to accomplish a common task (Cohen, 1984). It is also “the process of using power to obtain interpersonal influence” (Schermerhorn et al., 1985). Leadership requires influence and evocation to change the behavior of others in organizational settings and to attain established goals based on the agenda set. The core essence of leadership is “to get people to do what they do not want to do and like it” (Cohen, 1984). In this regard, leadership is the “ability to direct, motivate, encourage, and inspire others positively to a targeted end;” it connotes the ability to lead others (Olusoji, 2002).

Leadership functions through interactions between leaders and followers. It depends not only on leadership styles but also on the followership status held by different styles of leadership. Followership is contingent on, predicated, and determined by the leadership style used and can be voluntary (generated and grown intrinsically) or compelled/composed (generated and grown extrinsically; Akindele and Afolabi, 2013).

Voluntary followership grows under the permissiveness of a servant and ethical leadership and their humanistic philosophy and characteristics, while compelled followership usually develops under dictatorial leadership and its scientific management philosophy. The level of compelled followership depends on the follower's tolerance of indecent treatment by the leader. The bubble of compelled followership will eventually burst and lead to the extinction of dictatorial leadership, as well as change to a better leadership style (e.g., purposeful and humanistic leadership). This means that the type of followers a leader attracts is determined by the leader themselves. Facilitating effective followers becomes a *sine qua non* to any successful leadership. Fully developed, effective followers are the result of a leader's development of voluntary followership. Such followers are confident and committed to the performance initiatives of their leaders and make efforts to achieve organizational goals and mission.

### Leadership Theories: Background

Theories of leadership have been developed for over 100 years, and there have been three main phases. The first phase is dispositional (trait) theory (1920–1930). This theory of leadership is based on the characteristics of leaders and is used to predict leadership effectiveness. It states that effective leaders share some common personality characteristics, including personality (self-confidence and aggressiveness), intellect (intelligence, decisiveness, judgment, and knowledge), demographics (age, education, and socioeconomic background), social characteristics (sociability and cooperativeness), and task-related characteristics (achievement drive, initiative, and persistence).

Successful leaders definitely have some personality traits that are different from those of less effective leaders, and such traits are essentially seen as preconditions that endow people with leadership potential. Research shows that effective leadership occurs when there are publicly exhibited traits of strong cognitive ability (good judgment, strong analytical abilities, and conceptual skill), emotional maturity (well-adjusted and does not suffer from severe psychological disorders), integrity (trustworthy, reliable, and open), achievement drive (high levels of effort, ambition, energy, and initiative), and ethical decision-making (Olley, 2021). These traits are manifested because individuals believe them to be necessary for effective leadership. Leaders with such traits can initiate and maintain the structure of expectations for and interactions in the organization (Stogdill, 1974). While the traits theory is not a very fruitful approach to explaining leadership, it does have some implications that supervisors may want to be aware of in terms of potential leaders' strengths and weaknesses and that can thus be used as a reference for whether or not job candidates can develop their leadership qualities.

The second phase is the behavior theory of leadership, which dominated leadership theory from 1940 to 1970. Behavioral theories focus on what makes a good leader. One of the most popular behavioral theories is McGregor's Theory X and Theory Y. There are several basic principles of McGregor's theory. The central principle of an organization, which is derived from Theory X, is that of direction and control through the exerciser of authority, which has been called “the scalar principal.” The central principle that is derived from Theory Y is that of integration: creation of conditions such that the members of the organization can achieve their own goals best by directing their efforts toward the success of the enterprise. Accordingly, this theory posits the existence of three types of leaders: autocratic, democratic, and passive avoidant (Olley, 2021).

In the 1970's, the Contingency Model became the focus of leadership theory; it states that a group's performance will be contingent on the appropriate matching of a leadership style and the degree to which the organizational environment is favorable for the leader. This theory posits that leadership is contingent upon the situation, people, tasks, organization, and environmental variables. Also important is the degree to which the situation provides the leader with influence over their group members (Fiedler, 1967). The model assumes that several variables, such as position power, task structure, and leader–member relationships, shape leadership effectiveness. It also accounts for other factors, including the leader's and their members' intellectual abilities and technical qualifications, motivation of the group, and conditions of stress under which the group must operate.

Since the 1980's, theories on power and influence have been developed and have continued to advance. These approaches consider various ways that leaders use power and influence to achieve desired organizational outcomes. They include transactional (Bass and Avolio, 1993) and transformational (Bass, 1985; Shamir et al., 1993) approaches to leadership, authentic leadership (Luthans and Avolio, 2003), and ethical leadership (Brown et al., 2005). Other leadership approaches in contemporary organizations include neo-charismatic theories, servant leadership, adaptive leadership, respectful leadership, and transcendent leadership, among others. Fairholm (2010) divided such theories into five categories.

### Leadership Theories Relative to the Public Sector

This study focuses on leadership in public administration because the Chengguan belongs to the public sector, and all its members hold civil service positions. One of the primary leadership theories being introduced is the theory of leadership perspectives, which was posited by Fairholm (2010). This theory includes five leadership categories: scientific management, excellence management, values leadership, trust–cultural leadership, and whole-soul (spiritual) leadership. These five types function in a loosely hierarchical way, whereby scientific management is at the bottom, whole-soul leadership is at the top, and from the bottom-up, one set encompasses and transcends the other sets. Admittedly, the five components are very broad by nature, and they include a variety of theoretical domains and perspectives.

The scientific management leadership perspective emphasizes using a scientific management approach to maintain and promote productivity among employees; this includes the POSDCORB (plan, organize, staff, direct, coordinate, report, and budget) approach (Gulick, 1937). The excellence management type transcends scientific management by focusing on the so-called “excellence movement,” with systematic quality improvement as a primary process. Values leadership emphasizes that management objectives need to be achieved through shared values between leaders and followers, not merely through direction and control. It focuses on the leader's role in the leader–follower relationship. The trust–cultural leadership set emphasizes the shared culture and mutual trust between leaders and followers and recognizes that followers also play an important role in the relationship. The whole-soul leadership highlights the whole-soul nature of leaders and followers; when leaders treat followers as whole persons with emotions, knowledge, skills, and abilities that go beyond their job needs, they foster continuous growth, improvement, and self-leadership in an organization. On the basis of this theory, Fairholm (2010) proposed a leadership perspective model, provided a “holarchy” of the leadership perspective that includes a compilation of leadership elements, and produced a comprehensive view of the leadership phenomenon.

Compared with the theory of leadership perspectives, van Wart (2013) also summarized five well-recognized theories of leadership: classical management and role theory, transactional leadership theory, transformational leadership theory, horizontal or collaborative leadership theory, and ethical and critical leadership theory. These five broad theories can be individually linked to the theory of leadership perspectives and identify many widely agreed-upon and overarching insights on the topic.

Classical management treats leadership as equal to scientific management; organization goals are accomplished using human, financial, technological, and natural resources, which may require POSDCORB tools. Leaders are considered significant factors and, in some circumstances, the most important factor (Fernandez, 2005; Kaiser et al., 2008; Trottier et al., 2008). Leaders shoulder the responsibility of dividing and coordinating work in organizations when distractions, deterioration, and external challenges are constant and stable; more responsibilities and skills are needed in unstable times when distractions and challenges increase (Boin and Otten, 1996; Wheatley, 2006). The variety of demands and expectations of followers need to be constantly monitored by the leader (Moynihan, 2004).

As to a leader's role, Fernandez et al. (2010) identify five foci that serve as an important reference for this study. First, leaders need to lead task accomplishment by informing, communicating, and evaluating activities. Second, leaders need excellent leader–follower relationships to help followers easily achieve success. Third, leaders need to facilitate change by encouraging innovation and creativity. Fourth, leaders need to make sure that the public workforce largely represents the interest of the public. Fifth, leaders must lead with integrity not only by retaining standard virtues, such as honesty and selflessness, but also by discouraging and preventing unethical conducts, because authenticity has been claimed to be the “root construct” for other forms of “aspirational” leadership (Iszatt-White et al., 2021b).

Transactional leadership focuses on daily interactions between leaders and followers. It emphasizes the operational level, so it matches the street bureau's exercise well. Leaders need to meet followers' operational demands to give the followers the ability and qualifications to do their job. On the basis of contingencies, different leadership styles may be adopted to ensure that the requirements of the study are clearly presented and that clear paths are created for followers in achieving joint aims (House, 1996). In general, transactional leadership is suited more to a static public management environment.

Transformational leadership is about managing organizational change. Contemporary organizations become even more complex and chaotic, fomenting change, and dramatic change at times (Pollitt and Bouckaert, 2011). In this sense, the focus of change is more suited to a tumultuous world (Uhl-Bien et al., 2007).

Effective leaders not only need to ensure that things get done but also need to take the organization into the future. However, research indicates that transformational leadership alone does not produce success without effective management and transactional leadership skills, such as strategic planning, performance metrics, and collaboration with external resources (Kelman, 2011).

Horizontal leadership, or distributive leadership, is the idea that effective leadership can be attained by facilitating the use of “substitutes,” through clear protocols, unambiguous tasks, and effective frontline problem-solving, increasing the levels of training, and carrying out recruitment selection based on intrinsic satisfaction (Kerr and Jermier, 1978). However, to achieve the organization's joint goals, distributive leadership needs to be complemented by collaboration, which focuses on horizontal relationships across agencies (“networking”) and sectors (“partnering”). Only in this way can organizations effectively deal with problems facing contemporary leaders who need to enhance social integration, flatten organizations, and provide more organic structures that handle changes more fluidly (van Wart, 2013).

Ethical leadership includes three major pillars or concerns (Ciulla, 2004). The first is the intent of individuals. The second is selecting proper means for doing the right thing. The third pillar is selecting appropriate ends. Most would agree that all three pillars need to be functioning in order to facilitate effective and robust leadership (Ciulla, 1995). Ethical leadership also emphasizes authenticity and positivity, which are crucial points to being a leader. Authentic leaders are self-aware and focus on self-improvement (Avolio and Gardner, 2005; Gardner et al., 2005) in terms of their values, cognitions, and emotions. Positive means openness, transparency, and optimism (Luthans and Youssef, 2007; Norman et al., 2010); thus, leaders encourage openness, feedback, and effective communication by controlling their ego drives and defensiveness.

The specific aspects of leadership seem to be straightforward individually, but as leadership takes these aspects as a whole, it becomes complex and demanding. Also, leadership is dynamic and evolves along with specific and practical challenges over time. It is constantly being socially constructed, making it subjective and a moving target (van Wart, 2013). With reference to the leadership perspective model and relative mainstream leadership theories, we studied seven individuals' leadership behavior by examining their performance in a given city in China.

## Methodology and Materials

The primary step in the methodology is to develop a suitable leadership ontology with regard to this research. Leadership is a multifaceted and very complex subject of research (Oral, 2019); therefore, it demands a sound ontological stance to capture its essence. Drath et al. (1999) define leadership ontology as “the theory of entities that are thought to be most basic and essential to any statement about leadership.” There are three leadership ontologies: TRIPOD (leader–member shared goals), DAC (direction–alignment–commitment), and PVA (perception–value–achievement).

**Figure 1:**
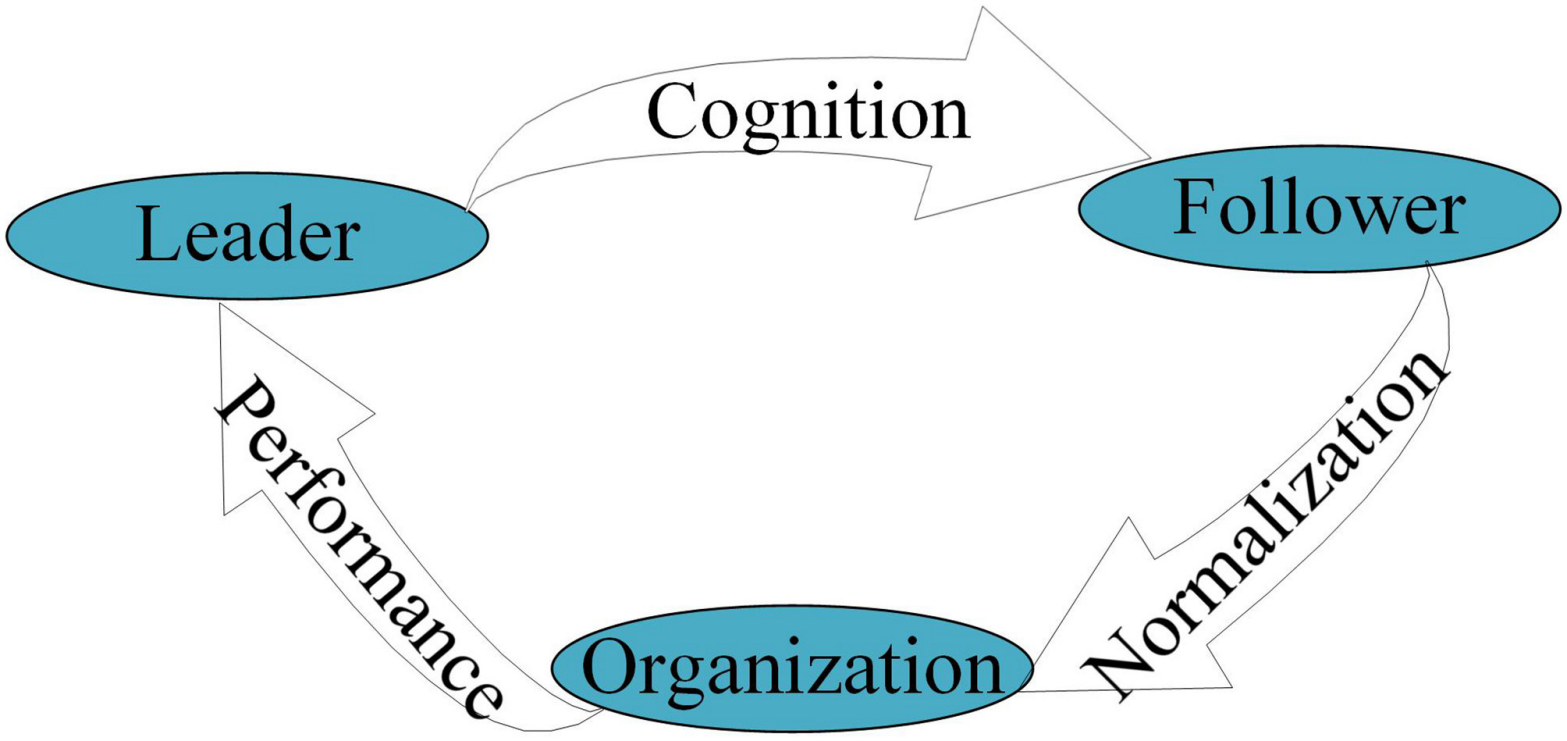
Leadership ontology Cognition–Normalization–Performance (CNP).

In association with existing leadership ontologies, and integrated with Chinese Chengguan practice, we posit a new Chengguan leadership ontology: CNP (Figure 1). Cognition reflects whether or not a leader perceives a situation they face in an organization (Chengguan Bureau, in this case), conceptualizes organizational and managerial issues, and formats their decision-making with reference to the leader's trait characteristics, experience, behavior assumptions, and so on, as well as external factors. Normalization means that a leader transfers their individual cognition and decision-making into organizational practices and routines, integrates it with colleagues and members, and establishes these as norms for an organization. Finally, performance refers to the organization's achievements and image as leadership is executed by interactions among internal and external stakeholders.

Based on this leadership ontology, an ethnographic case study in Z City Chengguan bureau between December 2001 and October 2019 was conducted. By applying this new leadership ontology to understand the behaviors of seven leaders in Z Chengguan bureau, ethnographic research was conducted to answer three sets of questions: (1) What shapes the leaders' cognition, including their traits and characteristics, behavior premises (McGregor's Theory X or Theory Y), career, and leadership experience, as well as leadership knowledge and training they have accumulated? (2) What action does the leader take for normalizing the Chengguan followers' work conduct (and how did they accomplish this)? Did the followers accept the leaders' instructions completely or conditionally? Or did they object to the leaders' directions and refuse to comply with the leader's intentions openly or privately? What is the major influence on interactions between the leaders and the followers? 3) What was the Chengguan organization's resulting performance following the normalization of the leaders decisions as a result of the leaders' cognition?

The three questions compose our analysis framework, and the answers to the above questions will provide meaningful guidance for the improvement of work in Chengguan. The ethnographic research enabled us to observe and identify beyond formal understanding different leaders (seven directors) executing their leadership decisions (Ybema et al., 2009). This case study presents the leadership styles and resulting work performance of seven directors of the Z Chengguan bureau in chronological order. By analyzing the interactions between the directors and the followers (e.g., acceptance/objection, obedience/resistance, identification/rejection, and internalization), this study explores the logic and meaning of the relationships between leadership styles and Chengguan's work performance.

### Data Collection and Analysis

Ethnography is not only a method but also an analytical perspective, and it is widely conducted in the field of organization and management research (Maanen, 2010). The ethnographic research conducted here follows a framework of zooming in and out (Nicolini, 2009). This framework provides a lens to study daily frontline exercises in the workplace, the interactions between leaders and followers, and the relationship between work performance and leadership styles. Zooming in comprises observing details and specific work activities to make sense of how this is achieved (Visser and Kruyen, 2021). In this case, we pinpointed staff members' law enforcement actions led by the respective directors, and we explored the bigger picture by observing the background context in which leadership was executed to better understand their interactions and the structural impacts.

The ethnographic fieldwork was conducted by the first author, who worked as a Chengguan staff member in this bureau from December 2001 to 2018, during which a total of seven directors had tenure. The first author participated in most of the organization's activities, and data were collected by observation; by note-taking, theough face-to-face interactions with the organization's members, including directors and staff; and with second-hand materials such as reports, and documents from the Chengguan bureau. In addition, some data were also employed from the first author's master thesis (Hua, 2005) and doctors' dissertation (Hua, 2015).

Data analysis was conducted simultaneously with data collection in an iterative cycle between collected data and relative theory (Klag and Langley, 2013). The first step was to classify the data into “seven leaders,” detailed descriptions, and narratives of sets of events (Langley, 1999). The identification process was conducted along with a broad-spectrum theoretical exploration (Gioia et al., 2013) of the concept of leadership. The final step was to further unravel the leadership performances by comparison, linking practice with theory.

Specifically, the study relies on the responsibility and code of conduct of the Z City Chengguan bureau as stipulated by the provincial government since the bureau's establishment in December 2001. These criteria gauge the Chengguan organization's performance and provide metrics for measuring performance in this research. In October 2018, the “Code of Conduct for Urban Management Law Enforcement” introduced by the Ministry of Housing and Urban–Rural Development of the People's Republic of China was implemented (the code's content is coincident to prior provincial regulations). The metrics of performance for the Z Chengguan bureau are displayed in Table 1.

**Table 1:** Metrics of Chengguan performance criteria.

Law enforcement discipline	Article 8: Urban management law enforcement personnel shall perform their duties on the basis of the legally prescribed authority, scope, procedures, and time limits and must not have the following conduct: (1) Selective law enforcement; (2) Threatening, abusing, or beating administrative counterparts; (3) Drinking alcohol during work or performing duties, or duties after drinking; (4) Tipping off administrative counterparts, concealing evidence, or excusing responsibility; (5) Retaliating against administrative counterparts; (6) Other conduct violating work discipline. Urban management law enforcement personnel shall recuse themselves when they have a direct interest in an administrative counterpart or a relationship that might affect fair law enforcement.
Case-handling norms	Article 12: Urban management law enforcement personnel shall lawfully carry out evidence prior registration, preservation, or sealing of sites and facilities, and seizure of property. Urban management law enforcement personnel shall properly store property that has been registered for preservation or seizure in advance and must not use, intercept, damage, or dispose of it without authorization.
Specifications for the use of equipment	Article 15: When urban management law enforcement personnel carry out law enforcement, they shall turn on audio-visual equipment, record the law enforcement process without interruption, promptly and completely store law enforcement audio-visual materials, and must not delete, alter, or transmit the original records.
Norms of manners and language	Article 20: When urban management law enforcement personnel carry out law enforcement, they shall behave in a dignified manner, have good posture, and behave appropriately and must not eat or fan while walking; they must not smoke in public places or other places where smoking is prohibited; they must not have their hands behind their backs, sleeve their hands, pockets, shoulders, arms, or waists, and laugh or make loud noises.
	Article 21: When urban management law enforcement personnel carry out law enforcement, they shall first raise their hands in salute to their administrative counterparts.
	Article 22: Urban management law enforcement personnel shall treat others with courtesy, use civilized and standardized language, and must not use vulgar, discriminatory, reprimanding, insulting, or threatening language against administrative counterparts.

## Findings

### Leadership Practices and Performance During the First Director's Tenure

The first director of the Z Chengguan bureau was L. H. Niu. He began his career as a soldier and then became a deputy monitor, monitor, deputy platoon leader, and platoon leader. Thereafter, Niu transferred from the army to work in the local government. Director Niu's leadership practices and his followers' performance are summarized here.

#### Lineup for Counting Every Day

As a veteran, Director Niu worshiped military life. Once he became a commander at the Chengguan bureau, he yearned to implement this style of leadership and announced his intention to apply “semi-militarized management.” Every morning, all bureau staff members paraded in an open space and formed a lineup for counting tasks, namely, standing, standing at ease, counting off, saluting, and so on, in accordance with the military procedure.

It must be noted that, although the bureau mandated all staff to participate in this lineup, those who had a post (e.g., the vice director, the section head, etc.) were exempted. Thus, it became a sign of class division in the bureau, as only the junior staff needed to line up for counting and were punished for their absence, while the heads and directors were exempted. This was discounted as a scaled-down version of militarization, and most staff members were therefore disgusted with it and were scolded for their disagreement. However, other government officials and the masses were initially pleased to see a “fresh start” and referred to those discounting it as *nao shao* (being silly).

#### Brick-Moving Management

Director Niu relished his “brick-moving management” theory in the first middle-level cadre meeting of the Z Chengguan bureau as follows:

When I was a commander of troops, if I found idle soldiers, I would instruct them to move bricks from the root of the south wall to the north wall, and then back from the north wall to the south wall, so the soldiers had something to do every day; otherwise, those 20-year-old energetic men may have brought trouble once they had time to think.

Overall, the core of the “brick-moving management” practice was to ensure that the team members had something to do at all times, so they would have no time to think or create. For personnel engaged in office work, Director Niu continuously had them draft all kinds of documents, regulations, and reports. Once the personnel finished these tasks and submitted them, the director usually reviewed them and evaluated them as unsatisfactory, and then instructed the personnel to rewrite the documents. This was usually repeated several times, and after the documents finally passed the evaluation, they were put on a shelf and forgotten. This whole process was conducted to keep the internal workers busy.

For the majority of personnel who worked in the field, Director Niu instructed them to always patrol the streets and conduct law enforcement activities. His motto was “farmers have to plant fields, workers do work in the workshop, and the Chengguan must patrol the streets.” While this statement was true, there were no work incentives or supervision mechanisms. Moreover, there were no resting places or living facilities for the Chengguan staff, and in the summer, with no heat protection measures, enforcement officers hid in shops to play poker or did other things and disappeared from the streets.

#### Working for Superior Inspections and Giving Notice to Vendors

Because of lack of internal motivation, external pressure became the main power to promote the work of the Z Chengguan bureau. The superior's inspections and some important city activities, such as local congress meetings or celebrity visits to the city, became good excuses for the director to arrange work and urge the enforcement officers to do their jobs.

When superiors inspected the effect of the management of Z city, Director Niu instructed the Chengguan staff to notify street vendors and merchants of the inspections and to instruct them to not come out or vend on the street. Thus, Chengguan's law enforcement became a conspiracy to fool the superiors, and it became a compromise between the Chengguan officers and the people they were intended to oversee, thus damaging the authority of law enforcement. This was not a long-term management solution for sustainable work. Ultimately, Chengguan's activities became “law enforcement theater” for the benefit of the superiors.

#### External Cowardice and Internal Chaos

As a newly established unit, the Chengguan bureau was despised not only by other government departments but also by the masses. A case in point is that, one evening, the plaque of the Z Chengguan bureau located on the gate of the office building was illegally removed. This was shameful, and it was suggested that the police be called to maintain authority. However, Director Niu directed all the staff members to not discuss it; rather than calling the police, he arranged for someone to make another plaque and hang it on the gate to “save face.”

The internal chaos was reflected by a series of fights among the staff, among the middle-level cadres, and even between the staff and vice directors; some examples of which are provided as follows: (1) the office administrator (Lv, Y.G.) fought with the head of the legal department (Jing, Y.W.); (2) one battalion member (Xie, J.H.) fought with a squadron leader (Fan, S.T.) because of work conflicts; (3) the head of the legal department (Jing, Y.W.) fought with the vice head of the legal department (An, D.H.); and (4) the deputy director (Zhao, X.D.) fought with a squadron leader (Fan, S.T.) during a dispute over an inspection issue.

The number of fights was so high that they cannot all be described. The Z Chengguan bureau's staff's dissatisfaction with Director Niu was evident; when the staff members met with Director Niu in the office or on the job, many of them ignored his presence. The director became aware of this and arranged for Deputy Director Zhao to notify all the staff members of the following: “When the Director comes to your office, everyone should stand up or give a nod as a signal to at least indicate his presence; this reflects minimum respect and courtesy.” However, this was not a solution to the root cause of the problem.

#### The Consequences of Director Niu's Leadership

When the Z Chengguan bureau was first established, the young staff members were very efficient; however, year after year, there was little upward mobility available to them. Therefore, the Chengguan staff members left the Z Chengguan bureau. In 2003, 6 of the 31 staff members were admitted to other civil service posts in the district, and the Z Chengguan bureau became a “talent transit point.”

In the second half of 2004, more than 20 demobilized soldiers who had transferred to the Z Chengguan bureau jointly wrote to the city's major leaders and complained about wandering the streets all day, not knowing what to do or how to do it. At the end of 2004, Director Niu resigned from his post, and a new director was appointed.

### Leadership and Performance During the Second Director's Tenure

The second director, L.J. Zhang (we will use “Zhang-L” thereafter), was the Deputy Director of the Z Municipal Public Security Bureau. Learning lessons from the first director, Zhang-L adopted a “strict management, strengthened supervision” management practice. The second director's leadership practices and his follower's performance are summarized as follows.

#### Fine Index

Because some of the staff members “did not know what to do or how to do it,” Zhang-L tailored a “fine index” for each squadron (i.e., each squadron had to collect a certain amount of fines). Therefore, all staff members who went out to conduct field work had something to do. Along with the stipulated fine index, an assessment procedure was also introduced, and the “bottom-out” policy was implemented.

#### “Tough Guy” Image

In one instance, to establish his authority in the bureau, Zhang-L punished three staff members and made an example out of others. Two squadron leaders (S.T. Fan and C.J. Li) and the vice head of the legal department (D.H. An) were removed from their posts and were forced to apologize during a conference with the entire staff for using litigant money for a private dinner. Consequently, many hecklers, such as those writing letters to the mayor to accuse the Chengguan Bureau's work content, were deterred.

In addition, Zhang-L set up a practice that required each staff member to pay a certain amount as a “cash deposit for regulation non-violation” every year. If a staff member did not violate the regulations of the bureau over the course of 1 year, the money was returned; otherwise, the money was confiscated.

Zhang-L adopted a practice of barbarism met with barbarism and established an inspector division in the bureau. He selected several staff members infamous for bad behavior to enter this division and allowed them to inspect the tasks of line-up for counting and other monitoring activities. To strengthen the system of line-up for counting, he required the deputy directors to watch the counting of staff members at the parade every morning.

#### “You Owe Me or I Owe You” Principle

The personnel affairs of staff management became a deal of selling commissions in the Bureau. If one staff want to take a middle-level commission, he had to buy the commission from this director, and further promotion also need the director's recommendation, which both need amply rewarded to him (in terms of money or other forms as most corrupt officials do in China). He placed the middle-level cadres and division heads as the cornerstone of his personnel management, and demanded that the members he promoted be thankful to him.

Where there is oppression, there is resistance, and this pressure placed on management gradually deteriorated. Primarily, issues arose within the leading group. For example, one deputy director was ordered by Zhang-L to apologize to an important litigant by providing them with a gift card, but the card was thrown out by the litigant and the apology was not accepted; thus, the deputy director was humiliated and dissatisfied with Zhang-L. Side effects of the middle-level cadre-centered work mechanism also appeared. The overemphasis on the importance of cadres alienated some staff members, and when some squadron leaders were besieged by vendors, the junior staff members stood by and did nothing.

Many junior staff members began to disappear after line-up for counting in the morning, and some took unpaid leave. Even some middle-level cadre members became worried about their future careers and began to ignore Zhang-L's instructions. His late efforts to set up “accounts” for street vendors and to let staff members “step patrol” the streets could not be promoted. Director Zhang later claimed by his own admission that he was “unable to bring people together.” At the end of 2008, Zhang-L was removed from his position.

### Leadership and Performance During the Third Director's Tenure

The next director was Z.F. Zhang (we will use “Zhang-Z” thereafter). His leadership can be summarized as “correction” to alleviate the external social dissatisfaction with the performance of the Chengguan, as well as to ease and coordinate internal conflicts among the staff members. The third director's leadership practices and followers' performance are summarized below.

#### Weakening the Practice of Line-Up for Counting

Absence from line-up for counting was no longer considered a “zero-tolerance” behavior, and it was not accompanied by any deduction of money or other direct punishments. In addition, the “cash deposit for regulation non-violation” practice was canceled; the staff members no longer needed to deposit money to the bureau.

#### Dilution of the Fine Index and Flexible Treatment of Assessments

To correct the unhealthy tendency of “He that serves God for money will serve the Devil for better wages,” Zhang-Z no longer adopted a rigid assessment regarding fines. Squadrons that did not achieve the minimum fine collection could still be treated well if they properly managed their designated streets. In addition, the inspection division was overhauled and adjusted; two members of the team who often “picked fights and provoked trouble” were removed from the division, and one of these two (Xu) was dismissed for violating the law.

#### Support Staff Members to Pursue Different Careers

Zhang-Z took a stance to support staff members who pursued their own careers and provided introduction letters, relevant certificates, and other resources. Nevertheless, the promotion channel of staff members was broadened; six township Chengguan stations were established, thereby opening up more middle-level positions for junior staff.

#### Set Up Trade Union Organizations and Enhance Welfare of Staff Members

The bureau began to conduct physical examinations for staff members once a year, issued birthday cards, and organized the middle cadres to study or travel for tourism, among other activities. In particular, a specialized institution for alcohol treatment was contacted to help an alcoholic staff member. Moreover, Zhang-Z did a great job of settling conflicts among the staff. For example, in one instance, two division heads (Liu and Jing) were engaged in a conflict at a table; Liu poured tea on Jing, after which the director curbed the conflict and then transferred Liu outside the bureau to work as an assistant in the Z Municipal Construction Bureau, thereby preventing the conflict from spiraling out of control.

All in all, although the third director did not remove some unreasonable practices and policies present in the Z Chengguan bureau, Zhang-Z did take many measures to reshape the organizational culture during his term in office. In 2010, he was transferred back to the Z Municipal Public Security Bureau as its director.

### Leadership and Performance During the Fourth Director's Tenure

The fourth director, Z.C. Dong, emphasized the needs and feelings of the staff members and ensured the availability of two-way communication between himself and the staff during his tenure. The fourth director's leadership practices and followers' performance are summarized as follows.

#### Abolishment of the Fine Index for Divisions

Compared with his predecessor's dilution of the fine index system, Director Dong completely canceled it. Instead, he emphasized management effectiveness in terms of the city's appearance. He adopted an approach to establish a city management grid, and each squadron's performance was assessed based on the city's appearance and living environment in each grid.

#### Outsourcing Some Operational Work Such as Painting Over Graffiti, to a Third Party

Director Dong outsourced some work (e.g., painting over graffiti) to prevent staff members from doing such work while wearing their uniforms. In this way, Chengguan's management image outweighed its working image.

#### Carrying Out Work Independently

Director Dong carried out work from the standpoint of the bureau's responsibility to ensure a superior arrangement of city management, not merely to pass inspections. He insisted that the Chengguan neither conspire with vendors and merchants nor carry out inspections or make special deployments or arrangements. When work tasks of higher-level arrangements obviously did not fall within the scope of the Chengguan work field, Director Dong refused to do the tasks.

#### Retaining the Practice of Line-Up for Counting

Director Dong alleviated the unfairness of some staff members exempted from the practice of line-up for counting. He personally attended this practice nearly every morning when he was in the office, and he asked the entire leading team, including the deputy directors, to attend.

Director Dong earned a good reputation from both his superiors and subordinates. Admittedly, some unfortunate events occurred as a result of the Chengguan's historically bad reputation. For example, one staff member, W.G. Li, was burned by a person in the street. Another employee, X.B. Tian, owed a gambling debt and left, and then died after leaving the bureau. It should be noted that the root of these problems was in the performance of the second director, L.J. Zhang, as many junior staff members found his cruelty unbearable and became passive to their work, thereby acquiring bad habits. Overall, the fourth director, Director Dong, played a strong role in the correction, adjustment, consolidation, and improvement of the organization.

### Leadership and Performance During the Fifth Director's Tenure

The fifth director, B. Gao, also came from the Z Municipal Public Security Bureau and filled the post in the latter half of 2013. Unfortunately, he died of illness on the job soon after (in early 2014); he, therefore, played a limited role in influencing the work of the Z Chengguan bureau and had a limited impact on the organization's culture. After he died, a deputy director, H.J. Zhang, took charge. The deputy director did not make substantial changes to the operation of the Z Chengguan bureau.

In the 2014 performance evaluation of the functional government departments of Z city, the Z Chengguan bureau was at the bottom of the ranking. In addition, in a televised political inquiry program (a new media program developed for the public to help them exercise their supervision rights and participate in local governance) held in July 2014, delegates of the people gave the lowest score to the Z Chengguan bureau based on city management-related problems. Thus, the Chengguan was dishonored, and the municipal government was not satisfied, so a new director was soon appointed.

### Leadership and Performance During the Sixth Director's Tenure

The sixth director, H.M. Han, came from the Z Municipal Construction Management Bureau. He was nominated by a municipal party committee and filled the post in early 2015. Director Han was familiar with the first director, Niu, and had been Niu's subordinate. The sixth director's leadership practices and followers' performance are summarized here.

#### Emphasis on the Inspection Division and Strengthening of the Practice of Line-Up for Counting

Director Han's management motto was “For administered objects, if you do not manage them, they will ignore you”. He reappointed the staff members present during the second director's term and gave them more power to inspect the junior staff members.

He also treated the practice of line-up for counting as the cornerstone of troop management. However, because most of the members were fed up with this practice, they could not keep abreast of the development of new forms of attendance (e.g., punching time clocks, accessing the attendance systems, etc.), and the practice incurred more complaints from junior staff members.

#### Fellow Villager-Based Clique Culture

Director Han was accustomed to forming small groups, and people from his village were deeply valued. One squadron commander, A.J. Liu, was removed from his middle-level position because of violation of bureau regulations; however, because Liu and Director Han were born in the same village, Han quickly reinstated Liu. In addition, some people were promoted and received benefits only as a favor from the director. This obvious clique culture was criticized by most staff members and sparked fury among the junior staff members.

#### Renaissance of the Working-for-Superiors Inspections

The municipal government had great expectations for the sixth director, and checks and inspections were more frequent. Director Han transformed this into a powerful weapon to urge the staff members to do their jobs. To prove that the Chengguan bureau was operating better under his leadership, Director Han reinstated the method of telling vendors and street merchants to coordinate to maintain the clean appearance of the city during city conferences or when higher-ups or celebrities were visiting.

### Leadership and Performance During the Seventh Director's Tenure

The seventh director, T. Zheng, came to the Z Chengguan Bureau after an accelerated promotion as a member of the Party Committee of the Z Municipal Public Security Bureau. The seventh director's leadership practices and followers' performance are summarized as follows.

#### Abolishment of the Practice of Line-Up for Counting

As of March 2017, the bureau had implemented the practice of line-up for counting for nearly 16 years, and the junior staff members consistently felt shamed and humiliated by this system. The seventh director engaged in a substantial amount of communication, listened to the views of the staff members, and finally decided to abolish this “iconic” product of the Z Chengguan bureau culture. This action received a positive response from both the staff members and other municipal departments. In fact, the third and fourth directors had also wanted to abolish this practice but did not act on these desires. Director Zheng garnered extensive support because of this reform.

#### Carrying Out a Rectification Movement for the Middle-Level Cadres

As mentioned, cliques and factions had become a problem in the Z Chengguan bureau; they obstructed new thinking and caused people to engage in internal strife rather than to think about adjusting their work situation. By the end of 2017, Director Zheng had taken a decisive action toward a dozen middle-level cadre members (heads of the squadrons or chief leaders in town stations) who were not in charge of their own affairs and let them engage in idle errands (e.g., reading magazines and newspapers, drinking tea, etc.), which hindered the bureau's development. Thereafter, he changed the law enforcement work style to the “outer block and inner leading” approach. He set up fixed sales sites over the urban area of Z city for mobile stalls and vendors, thereby solving the chaos of disorderly selling by vendors.

#### Normalization of the Z Chengguan Bureau to a Regular Municipal Unit

Since the establishment of the Z Chengguan bureau, successive directors have more or less recognized its particularity, i.e., it was a new unit, it had arrangements with vendors and street merchants, it was a type of street bureaucracy, a large portion of the staff was demobilized military personnel, and some of the deputy directors transferred to civilian life from the army. However, the more emphasis that was placed on its particularity, the less it was recognized. Therefore, Director Zheng promoted the normalization of the Z Chengguan bureau, i.e., by adopting common checks on work attendance with other municipal departments, coordinating with the organizational department of the Communist Party municipal committee to make personnel arrangements, and sending middle cadres to communities and other municipal departments for work. Gradually, the Z Chengguan bureau cadres became recognized as “brother units.”

The leadership, practices, and work performance of the seven directors are summarized in Table 2.

**Table 2:** Analysis of the leadership, practices, and work performance of the seven directors.

**Leader**	**Followers composition and situation**	**Leader cognition**	**Follower normalization**	**Performance appraisal**
First director (December 2001–May 2005) ***Experience management***	December 2001: 2 vice directors and 8 clerks were transferred from the municipal government to create the bureau. May 2002: 20 college students were recruited as clerks. June 2003: 6 demobilized soldiers entered the bureau. May 2003: 15 college students were recruited as clerks. July 2013: 6 clerks were transferred to other departments of the municipal government through an exam. December 2014: 30 demobilized soldiers entered the bureau; when the first director stepped down, around 75 clerks were present in the bureau.	Trait characteristics: 1. No confidence, servile to external pressure 2. Lacks decisiveness, judgment, and knowledge 3. Non-sociable and non-cooperative 4. No achievement drive; work motivation comes from superior inspection (no work plan, no arrangement, work at random) McGregor's Theory X: Staff must be kept busy; otherwise, they will introduce trouble; emotional connection between the director and staff members is not viable	1. Line-up for counting 2. “Brick-moving management” 3. Worked under external pressure, colluding with vendors and meeting the superior's inspection requirements 4. Connivance of fights among staff members	Municipal government appraisal was poor. Most clerks worked with a go-slow attitude. Some clerks escaped from the bureau; some of them accused the mayor. Maxwell's five levels of leadership: 1. Position
Second director (May 2005–August 2009) ***Scientific management***	May 2005: 15 college students were recruited as clerks Normal staff entered or exited in single figures, and by the end of August 2009, the total clerks of the bureau were 100 or so. The main body still was college students; several troublemakers among the 30 demobilized soldiers consisted of a new section—inspection. There were no open objections from the clerks. The second director adopted a “carrot and stick” approach to treat the two main groups of clerks: it sets up some mid-level cadre positions to solicit potential troublemakers from among the demobilized soldiers, but it also intimidated the college students.	Trait characteristics: 1. Self-confidence and aggressiveness 2. High intelligence and achievement drive 3. Two-faced person, created a “tough guy” image, but forcible-feeble hypocrisy McGregor's Theory X: People were cowardly bullies; if you do not bully others, others will bully you 1. Executed power as trading (“you owe me, or I owe you”), e.g., use official positions in exchange for benefits, etc. 2. Position-oriented management practice; “at his wit's end when staff members do not care to get a position”	1. Strengthened the practice of line-up for counting 2. Established an inspection division to strengthen internal supervision 3. Set up a “fine index” for each law enforcement division 4. Developed a “one-day code of conduct” for staff members 5. Made a “cash deposit for regulation non-violation” policy	Municipal government appraisal was good at the beginning and poor finally: the director confessed that he was “unable to bring people together.” at the end. Maxwell's five levels of leadership: 1. Position+ 3. Production (to some extent)
Third director (August 2009–April 2011) ***Values leadership***	Six town law enforcement stations were approved, and around 20 clerks were enlisted. Most are demobilized soldiers and a few were transferred from other departments of municipal governments. About 120 clerks were in the bureau at this point. College students and demobilized soldiers had equal weight.	Trait characteristics: 1. Self-confidence, decisiveness 2. Sociability and cooperativeness 3. Achievement drive McGregor's Theory Y: “Personal development/growth is the premise of collective development” Paid attention to motivation, including material factors, and career prospects for staff members Emphasized task completion and paid attention to higher-level arrangements; attempted to solve conflicts between middle-level cadres	1. Weakened supervision of the practice of line-up for counting 2. Diluted the fine index assessment to the subordinate division 3. Set up labor unions; increased welfare for staff members, e.g., medical examinations 4. Regularly sent middle-level cadres to go out to visit, etc.	Municipal government appraisal was good promotion to be a commissionar of Z Public Security Bureau. Maxwell's five levels of leadership: 3. Production
Fourth director (April 2011–June 2014) ***Ethical (spiritual) leadership***	Personnel composition was constant. The bureau started to employ auxiliary clerks to assist work. The auxiliary clerks (first batch is 20 people; increased rapidly to around 100) were not the formal staff; they signed a contract with municipal government personnel bureau and worked under the arrangement of the Chengguan bureau.	Trait characteristics: 1. Self-confidence 2. High intellect, good judgment 3. Sociability and cooperativeness 4. Initiative and persistence McGregor's Theory Y: Easy going to the staff members, flexible to the arrangement of work, respect for the deputy director's opinions, and listened to staff members 1. Advocate self-restraint, self-management, and self-conscious compliance incentive 2. Open, face to face, QQ, WeChat, communication with staff members	1. Abolished the fine index for subordinate divisions 2. Established an outsourcing mechanism; staff members no longer needed to paint over graffiti 3. Promoted grid management; constructed a new grid team to assist the law enforcement	Municipal government appraisal was good at first, then mediocre. Maxwell's five levels of leadership: 4. People development
Fifth director	Regular personnel flow without much change	***Accidental death*** (One vice director acted as a deputy)	***Too short to be neglected***	***Poor***
Sixth director (May 2015–June 2017) ***Experience management + scientific management***	Several batches of demobilized soldiers (Chengguan bureau was the main venue for their resettlement) enlisted with the bureau, now numbering 150.	Trait characteristics: 1. Self-confidence, high intellect 2. Non-sociable and non-cooperative 3. Two-faced person: servile to superiors, harsh to subordinates McGregor's Theory X: “For administered objects, if you do not manage them, they will ignore you” 1. Build “fellow-villager-based clique” 2. Renaissance of working for superior inspections, indifference to followers' demands	1. Strengthened the practice of line-up for counting 2. Reused the inspection division to strengthen internal inspections 3. Restarted the model of working for superior inspections and giving notice to vendors	Municipal government appraisal was mediocre Maxwell's five levels of leadership: 1. Position+ 2. Persimmison (part of the followers)
Seventh director (June 2017–June 2020) ***Ethical (spiritual) leadership***	40 Municipal Patrol Brigade clerks, who belonged to the Municipal Public Security Bureau before, were allocated to Chengguan bureau. Most of the 40 Municipal Patrol Brigade clerks were demobilized soldiers; at this time, the portion of the demobilized soldiers accounted about 60% to the total numbers of clerks. Clerks united to push the seventh director abolish the line-up for counting practice, as well as address other honorable issues for clerks… As of June 2020, there were 186 members in the Z Chengguan bureau.	Trait characteristics: 1. Self-confidence, high intellect 2. Sociable and cooperative 3. Achievement drive, initiative McGregor's Theory Y: “whoever has the skill and ability can play” 1. Decisive and responsible 2. Innovation in work cognition 3. More democratic during work (listened to staff members' opinions)	1. Abolished the practice of line-up for counting 2. Abandoned the fine index for subordinate divisions 3. Carried out a rectification movement and changed the thinking and priorities of work (e.g., set up fixed sale sites for mobile stalls and vendors)	Municipal government appraisal was good the Director promoted to be a director-equivalent leader of Z Public Security Bureau Maxwell's five levels of leadership: 3. Production 4. People development

*The work performance is according to the municipal government's assessment of its departments: percentage ranking 99–90 is excellent, 90–70 is good, 70–10 is mediocre, and 10–0 is poor. The percentage range may change slightly in different years*.

*The Maxwell style levels of leadership are (1) position, (2) permission, (3) production, (4) people development, and (5) pinnacle*.

Their performance was evaluated by the municipal government on the basis of the “Code of Conduct for Urban Management Law Enforcement.” In addition to the municipal government appraisal of Chengguan's clerks and their director, Maxwell' five levels of leadership were also used for supplemental evaluation:

1. Position: people follow because they have to.
2. Permission: people follow because they want to.
3. Production: people follow because of what you have done for the organization.
4. People development: people follow because of what you have done for them personally.
5. Pinnacle: people follow because of who you are and what you represent.

The first director promoted a semi-militarized form of management. He thought his subordinates were avoiding work, so he tried his best to make them busy but failed to achieve their basic need for autonomy and competence (Deci and Ryan, 2000). Thus, the staff members found ways to counteract the silly and ridiculous practices (e.g., the “brick-moving management” theory and line-up for counting), and the results were disastrous for the organization. He also lacked the very important interpersonal motivation to communicate to followers, as he stated that an emotional connection between a leader and their followers is inappropriate. All of these opinions are in conflict with the modern leadership theory. A leader's mood in an organization does have some effects on the group (Cote and Saavedra, 2005). George (2009) articulated that “leadership can be perceived as an emotion-laden process with emotions entwined with the social influence process.” Iszatt-White et al. (2021a) proposed that emotional labor is integral to a leader's role.

The second director transcended the first by pretending to be a “tough guy” and used many approaches and tools of scientific management to treat the staff members fiercely, i.e., setting up a “fine index,” formulating the “cash deposit for regulation non-violation” policy, and so on. In this circumstance, even though he had the competence of scientific management skills, his leadership philosophy of executing power as a form of trading (“you owe me or I owe you”) terminated his leadership position, and he later felt that “I was unable to bring people together.” His two main tactics were position-oriented management practice (carrot) and “punish someone as a warning to others” (stick), but he failed because he was outwardly strong and inwardly weak. The sixth director, who can be placed somewhere between the first and second directors, also built “fellow villager-based cliques” and did not achieve an effective work performance. The abovementioned directors have in common an egotistic streak, which contradicts the well-recognized leadership theory that responsible and effective leaders always put the needs of subordinates first (Cooper and Wright, 1992; Giacalone and Jurkiewicz, 2003).

In contrast, the third director mainly adopted a transformational (values) form of leadership, while the fourth director exerted a transformational (trust–cultural) form of leadership, and the seventh director presented an ethical (spiritual) form of leadership. Among these directors, the seventh director seems to have achieved the most success. He abolished the practice of line-up for counting, canceled the fine index system, and adopted an “outer block and inner leading” approach. With the support of staff members, he created opportunities for Chengguan to achieve a successful future.

It is observed that the third, fourth, and seventh directors exerted more competence in teamwork, interpersonal skills, and communication. They also presented relationship-oriented leadership in terms of coaching and providing followers with the necessary information and the resources needed to carry out their job duties (Arnold et al., 2000; Ahearne et al., 2005; Park and Hassan, 2018). However, all the directors failed to practice role-modeling coaching and teaching (Schein and Francisco, 1992), and none adopted empowering practices, even though doing it may have improved public employees' job satisfaction and organizational commitment according to Western scholars' research (Hassan et al., 2013; Fernandez and Moldogaziev, 2015; Kim and Fernandez, 2017).

## Discussions and Conclusion

Currently, street-level practitioners like Chengguan across domains and countries have been increasingly brought into focus. Their dismal public image and poor job performance need to be solved by novel ways of working and unique solutions (Johansen and van den Bosch, 2017; Kruyen and van Genugten, 2017; Rutz et al., 2017; Sabel et al., 2017; Raaphorst and Loyens, 2020). This can be achieved by improvement of leadership under the principle that the “success or failure of every human endeavor depends solely on the kind of leadership available for such endeavor” (Youth Heritage Development Centre, 2009). Since leadership is required in public administration to resolve its inherent imperfections (Behn, 1998), the kind of leadership that public administrators should be practicing needs to be examined, including their initiative, motivation, inspiration, and other qualities (Terry, 1995). This article contributes to further understanding this issue by conducting ethnographic research and discussing both research and practical implications (Visser and Kruyen, 2021).

### Theoretical Implications

We contribute to the study on leadership theories by developing a new leadership ontology grounded in a strong methodology and the new concept of CNP. On this foundation, by ethnographic research to analyze the leader's cognition, followers' normalization, and organization's performance, several findings were attained.

First, the leader's cognition, as the source of leadership, involves many factors. A superior can avoid mistakes by taking care to address some issues in advance (e.g., their leadership experience and traits). A consensus has been reached that governments should be cautious in assigning a demobilized cadre member as the principal person responsible for an organization in China's government, because such an individual is accustomed to obeying orders and some (e.g., the first director) indulge in military life, so that they may exert their leadership with misguided assumptions. This may lead to a disaster for the followers and organization. While moral characteristics have a greater influence on the appraisal of theleader as competent, moral obscenities are more dangerous to an organization.

Second, in the leadership normalization process, followers can exert a positive and strong influence to affect the leadership, as well as to shape the organization's changes, but only when their relative strength is not weakening. For example, in 2005, 30 new demobilized soldiers came to the bureau, expressed their dissatisfaction with the first director, and wrote letters to the mayor; this caused the first director to step down. To some extent, this also benefited the other followers. As college students were the main staff during the 2011–2015 period, they were submissive and not united; they only objected to the director in passive ways such as slipping away from work and escaping to play cards (poker) during work time. The first director kept a lid on things. The watershed was when the demobilized soldiers wrote open letters to the mayor and complained about their work conditions and job duties. The director then lost control of the bureau and changed the organization to a great extent. Similarly, in 2017, when 40 new clerks came to the bureau, “the influential increment over and above mechanical compliance with the routine directives of the organization” (Katz and Kahn, 1978), they exerted great influence on the seventh director, which resulted in the leader abolishing the 16-year practice of line-up for counting. This approach also contributed to the organization going beyond the required performance and realizing more fully the potential of a given position for organizational influence.

Third, the performance of the organization interacts with the organization's context (Avolio, 2007). The leadership context is characterized as a superior attitude and social concerns about an organization. The external environment, known as the leadership situation, lies outside the leadership ontology and plays a decisive role in the judgment of leadership. Contingency theory addresses this point, as the context has certain effects on leaders and followers who are embedded in it.

Fourth, we employed an integrated leadership model to analyze leadership effectiveness on the basis of existing organizational leadership approaches. In this regard, we observed and identified factors beyond prior formal understanding. Compared with Fairholm's leadership perspective model, where scientific management lies at the bottom, this study found that, below scientific management, there is another leadership perspective: experience management or traditional management. This happens when someone is placed in a leadership position without scientific management knowledge and skills, such as the first director of the Z Chengguan bureau. He knew little about planning, organization, coordination, and supervising, and he wielded power by relying on his experience and instructions and orders from his superior. The practices of line-up for counting and brick-moving management came from his military career, and he encouraged colluding with vendors to meet the superior's inspection requirements. Such cases are not rare in some organizations in China, and in basic, small cases, it works well. Thus, a proposed theory of leadership perspectives derived from Fairholm falls into a new set of four categories: experience management (traditional management), scientific management, values leadership, and ethical (spiritual) leadership.

With reference to van Wart (2013) five categories of leadership, horizontal leadership may not serve well for rule-bound and hierarchical organizations like Chengguan. Under an authoritarian regime, together with traditional Chinese culture, the horizontal leadership approach seems to have no space in China's organizational management. An ideal form of leadership should liberate followers to build community and promote committed stewardship while modeling a service orientation (i.e., to be servant leaders), thus developing and enabling individual wholeness in an organization and building moral standards to facilitate people's liberation in the context of self-sustainable development and service delivery.

### Practical Implications

First, it is necessary to improve the leader's selection and training. When a leader is always stagnated and has no vision, like the first director of the Z Chengguan bureau, they will resist, be reluctant to, or respond slowly to change, and the consequences will be very serious (Stare, 2006). Therefore, selection of a leader is important. McGregor's Theory X states that superiors should be cautious when appointing someone to a leadership position. This is because “those firmly committed to their own ideas are not necessarily good change leaders” (Fullan, 2002). With that in mind, more attention must be paid to individual personalities and traits by investigating the candidates' career paths, educational levels, and motive profiles during the selection process.

Specifically, the Z Chengguan bureau should select a director from the internal membership instead of appointing one from the outside. In addition, leadership training should be conducted regularly to help good leaders improve and to rectify some bad ones.

Second, position- and treatment-oriented leadership approaches (e.g., the second director's leadership, an alienation to scientific management in this study) need to be transformed into people-oriented leadership. In the case of the Z Chengguan bureau, all the incentive and control practices were centered around “position and treatment;” the staff members cared about becoming officials, not about responsibility. To change this situation, a leader's bureaucratic philosophy must transform to a sense of responsibility.

Third, leaders should be encouraged to learn ethical (spiritual) leadership skills to be a whole-soul leader. While scientific management is the base of leadership, ethics is the core, which covers and transcends from values to trust and cultural leadership. Ethical leadership has a number of dimensions that are related to the wholeness of individuals in a society. The first dimension is honesty. Some directors in this research presented an empty slogan such as “enormous capacity for hard work, combat, and endurance,” which is contrary to this principle. The second dimension is trustworthiness; leaders should not “say one thing and do another.” The third dimension is fairness; the failure of this is demonstrated in a case study on the first, second, and sixth directors who divided staff members into many classes and treated them differently. The fourth dimension is conscientiousness. At a basic level, conscientiousness means working earnestly and deservedly; at a higher level, conscientiousness means striving for excellence (Dull, 2009) to fulfill a duty, which includes respect for professional norms, working rules, and laws (Menzel, 2007; Sergiovanni, 2007).

Fourth, as to the improvement of the leadership environment (context), the superior (the municipal government, in this case) should limit interference in the bureau's operation. On the one hand, avoiding excessive interference in internal affairs of the organization's work affects the leaders. For example, the fourth director was very decisive and always insisted on working independently, regardless of the unreasonable demands and work (e.g., house demolition) requested from the city government. On the one hand, he urged the organization to set up a labor union, youth federation, women's federation, and so on to strengthen the followers' unity and coherence. Thus, it is possible to achieve an incremental influence to balance the authoritarian power of the director. In addition, to supervise the problem with regard to the organization's operation and performance, it is necessary to take measures in time on the one hand and give the leader, the followers, and the organization a flexible space to adapt on the other hand.

Admittedly, our study has several limitations that should be noted. One limitation comes from the case study and ethnographic research method. The ethnographic methodology has advantages in collecting different types of data from diverse sources: documentation, archival records, direct observation, physical artifacts, and interviews. This results in a fuller picture of the phenomenon than would have been achieved otherwise. However, analysis of data is not an exact science; records of data are not systematically created, which may not sufficiently convince the audience of their accuracy. Another limitation is the integrated leadership approaches adopted. While this study starts with the CNP leadership otology, we attempted to integrate trait theory, behavior theory, and power and influence theories to analyze the leaders' cognition, followers' normalization, and organization's performance. These approaches cannot test all components of the conceptual model, i.e., we omitted physiological (appearance, height, and weight) and demographic (age, education, and socioeconomic background) characteristics and focus on personality, intellectual, task-related, and social characteristics. With regard to behavior theory, we only employed McGregor's Theory X and Theory Y. In addition, the leadership styles of six of the directors fall into four categories: traditional (experience) management, scientific management, values leadership, and ethics leadership. Other leadership theories, such as servant leadership (Greenfield, 2007) and empowering leadership (Arnold et al., 2000), are neglected. If these are considered, more profound results may be obtained.

Finally, there is one limitation of this study that future works may explore: the boundaries of leadership and its context. The leader, followers, and organization are the internal field of leadership, while the external field mainly refers to the organization's superior and the public, who are not independent of the internal field. This is evident in contingency theories of leadership, although the argument is still weak (Avolio et al., 2003). In this study, we did not examine whether the work context can “activate” the expression of a given trait of a leader; in fact, the leaders' traits and leadership behaviors are also influenced by the structure of work.

Overall, this research is in accordance with prior leadership theories on understanding leadership; moreover, it adds new knowledge to the Chinese context. For future studies similar to this one, the presrent study will contribute to knowledge-building in other fields, especially in street-level bureaus and hierarchical organizations. As such, an ethnographic case study cannot generalize our results to other public service professions. The leadership perspectives may be reinterpreted, and the specific model and competencies needed by public sector leaders may also be reevaluated (van Wart, 2013).

## Data Availability Statement

The original contributions presented in the study are included in the article/supplementary materials, further inquiries can be directed to the corresponding author.

## Author Contributions

HL contributed to the conception of the study and writing of the manuscript. YL contributed to the analysis and manuscript preparation. All authors contributed to the article and approved the submitted version.

## Conflict of Interest

The authors declare that the research was conducted in the absence of any commercial or financial relationships that could be construed as a potential conflict of interest.

## Publisher's Note

All claims expressed in this article are solely those of the authors and do not necessarily represent those of their affiliated organizations, or those of the publisher, the editors and the reviewers. Any product that may be evaluated in this article, or claim that may be made by its manufacturer, is not guaranteed or endorsed by the publisher.
